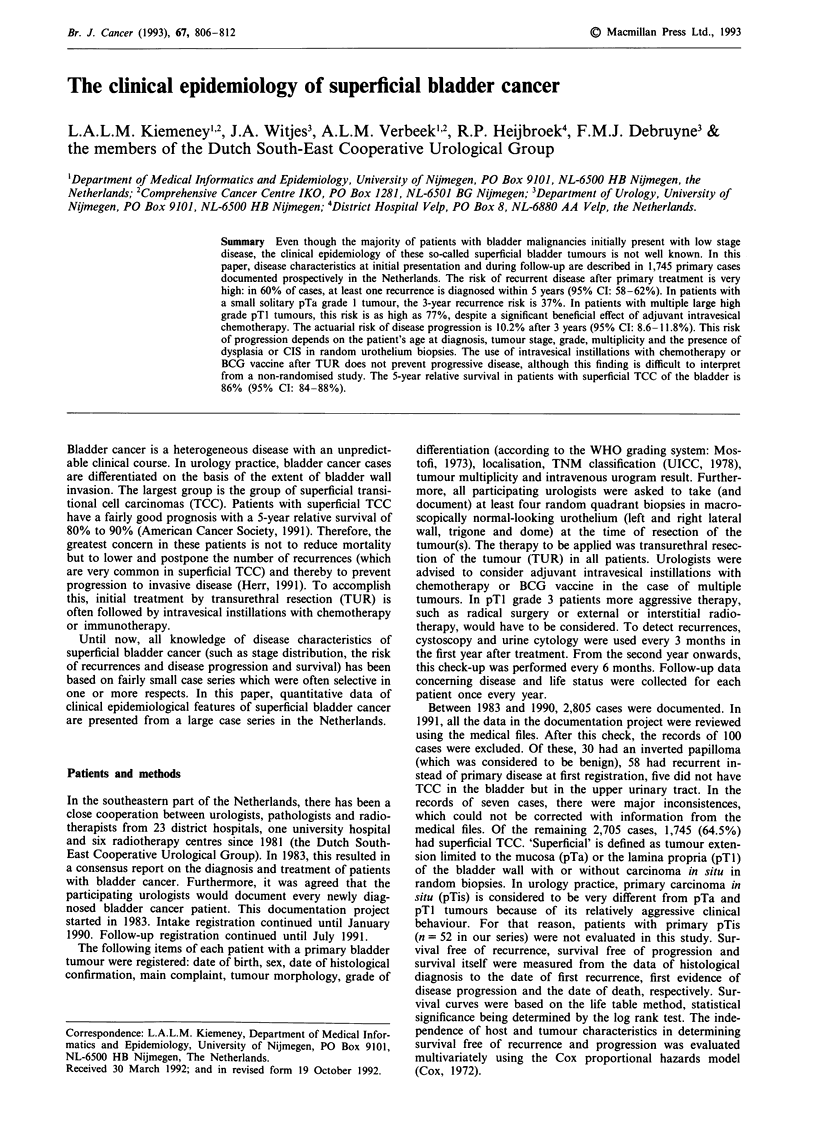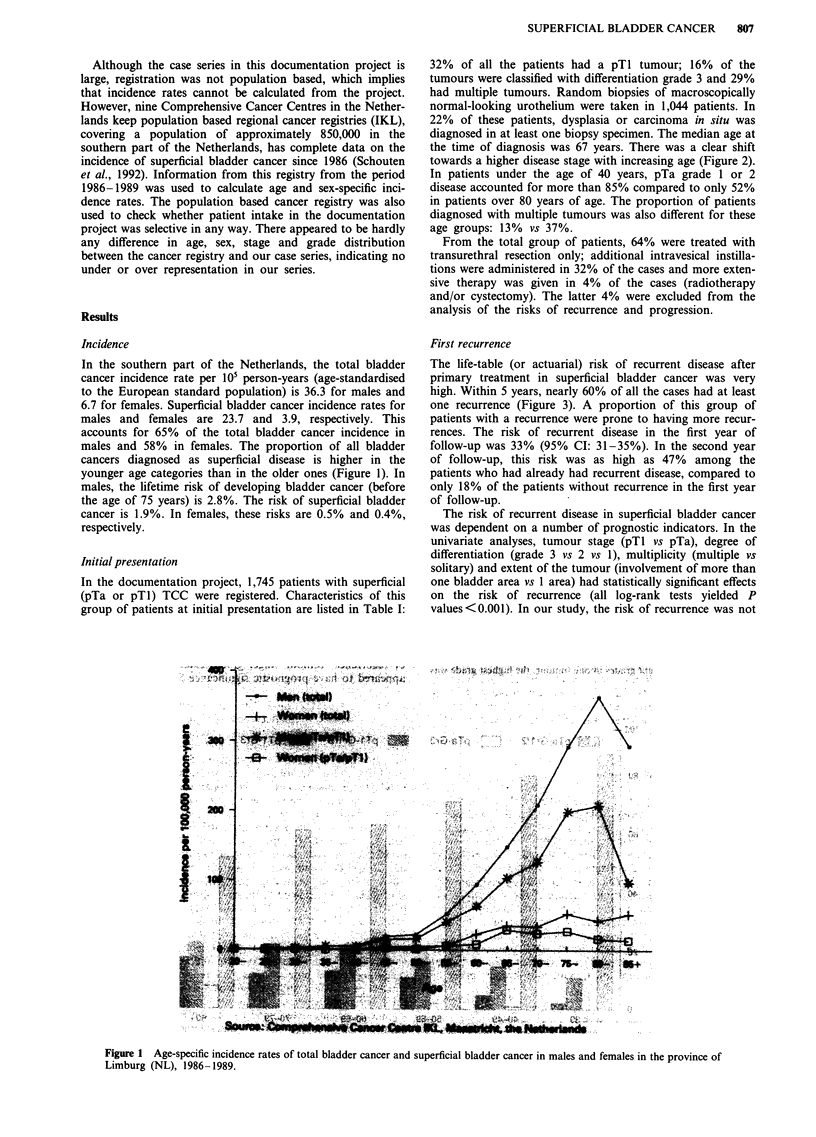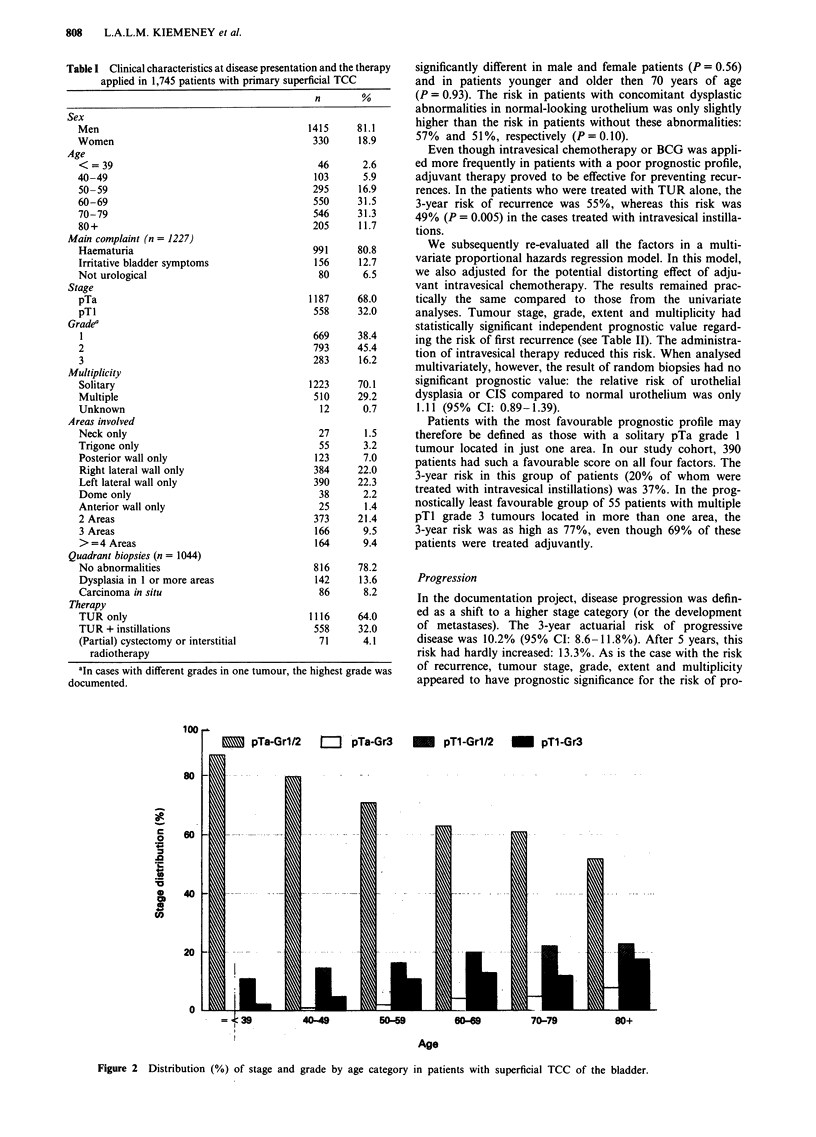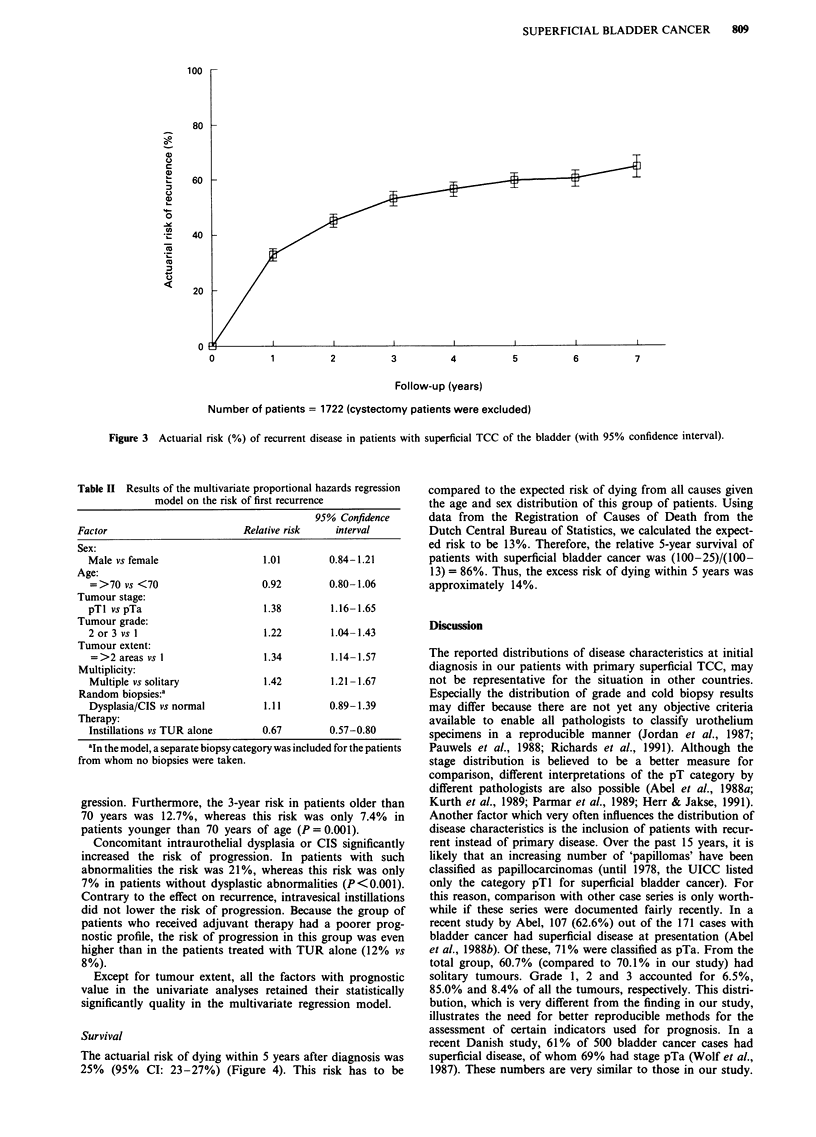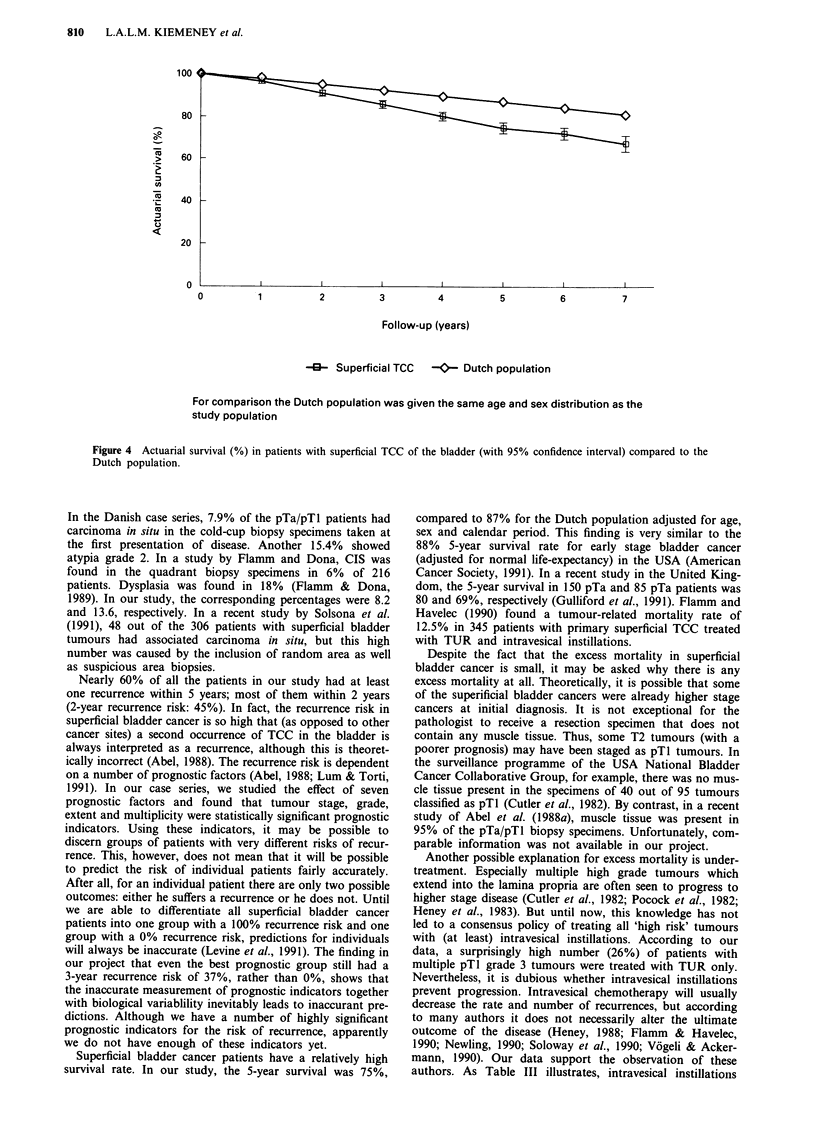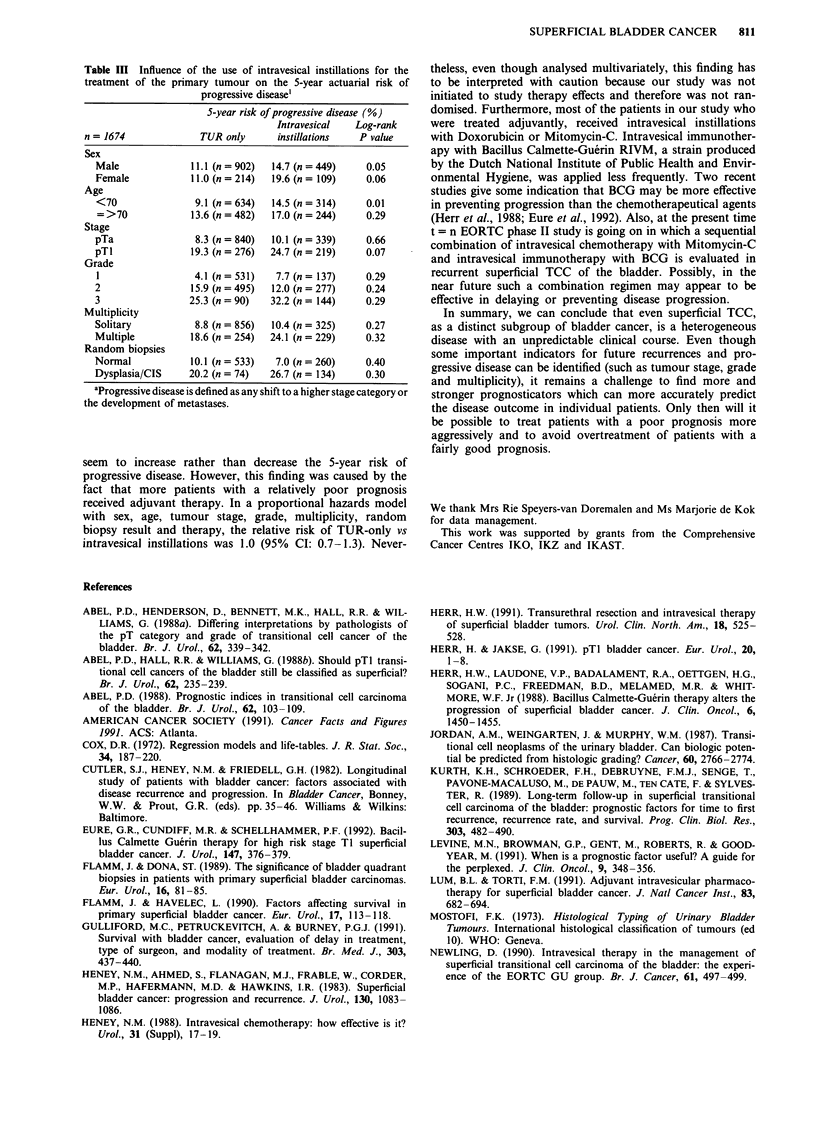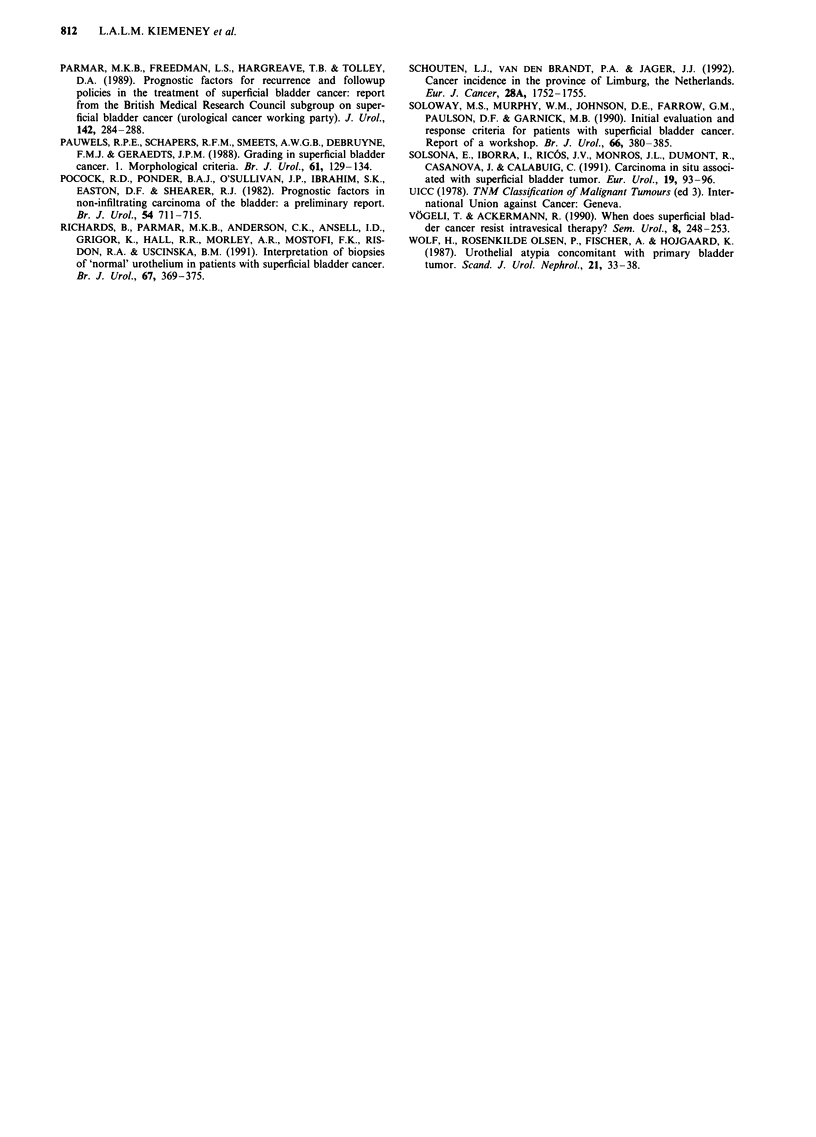# The clinical epidemiology of superficial bladder cancer. Dutch South-East Cooperative Urological Group.

**DOI:** 10.1038/bjc.1993.147

**Published:** 1993-04

**Authors:** L. A. Kiemeney, J. A. Witjes, A. L. Verbeek, R. P. Heijbroek, F. M. Debruyne

**Affiliations:** Department of Medical Informatics and Epidemiology, University of Nijmegen, The Netherlands.

## Abstract

Even though the majority of patients with bladder malignancies initially present with low stage disease, the clinical epidemiology of these so-called superficial bladder tumours is not well known. In this paper, disease characteristics at initial presentation and during follow-up are described in 1,745 primary cases documented prospectively in the Netherlands. The risk of recurrent disease after primary treatment is very high: in 60% of cases, at least one recurrence is diagnosed within 5 years (95% CI: 58-62%). In patients with a small solitary pTa grade 1 tumour, the 3-year recurrence risk is 37%. In patients with multiple large high grade pT1 tumours, this risk is as high as 77%, despite a significant beneficial effect of adjuvant intravesical chemotherapy. The actuarial risk of disease progression is 10.2% after 3 years (95% CI: 8.6-11.8%). This risk of progression depends on the patient's age at diagnosis, tumour stage, grade, multiplicity and the presence of dysplasia or CIS in random urothelium biopsies. The use of intravesical instillations with chemotherapy or BCG vaccine after TUR does not prevent progressive disease, although this finding is difficult to interpret from a non-randomised study. The 5-year relative survival in patients with superficial TCC of the bladder is 86% (95% CI: 84-88%).


 © Macmillan Press Ltd., 1993

### The clinical epidemiology of superficial bladder cancer

L.A.L.M. Kiemeney[1,2], J.A. Witjes[3], A.L.M. Verbeek[1,2], R.P. Heijbroek[4], F.M.J. Debruyne[3] & the members of the Dutch South-East Cooperative Urological Group

[1]*Department of Medical Informatics and Epidemiology, University of Nijmegen, PO Box 9101, NL-6500 HB Nijmegen, the Netherlands;* [2]*Comprehensive Cancer Centre IKO, PO Box 1281, NL-6501 BG Nijmegen;* [3]*Department of Urology, University of Nijmegen, PO Box 9101, NL-6500 HB Nijmegen;* [4]*District Hospital Velp, PO Box 8, NL-6880 AA Velp, the Netherlands.*

**Summary** Even though the majority of patients with bladder malignancies initially present with low stage disease, the clinical epidemiology of these so-called superficial bladder tumours is not well known. In this paper, disease characteristics at initial presentation and during follow-up are described in 1,745 primary cases documented prospectively in the Netherlands. The risk of recurrent disease after primary treatment is very high: in 60% of cases, at least one recurrence is diagnosed within 5 years (95% CI: 58-62%). In patients with a small solitary pTa grade 1 tumour, the 3-year recurrence risk is 37%. In patients with multiple large high grade pT1 tumours, this risk is as high as 77%, despite a significant beneficial effect of adjuvant intravesical chemotherapy. The actuarial risk of disease progression is 10.2% after 3 years (95% CI: 8.6-11.8%). This risk of progression depends on the patient's age at diagnosis, tumour stage, grade, multiplicity and the presence of dysplasia or CIS in random urothelium biopsies. The use of intravesical instillations with chemotherapy or BCG vaccine after TUR does not prevent progressive disease, although this finding is difficult to interpret from a non-randomised study. The 5-year relative survival in patients with superficial TCC of the bladder is 86% (95% CI: 84-88%).

Bladder cancer is a heterogeneous disease with an unpredictable clinical course. In urology practice, bladder cancer cases are differentiated on the basis of the extent of bladder wall invasion. The largest group is the group of superficial transitional cell carcinomas (TCC). Patients with superficial TCC have a fairly good prognosis with a 5-year relative survival of 80% to 90% (American Cancer Society, 1991). Therefore, the greatest concern in these patients is not to reduce mortality but to lower and postpone the number of recurrences (which are very common in superficial TCC) and thereby to prevent progression to invasive disease (Herr, 1991). To accomplish this, initial treatment by transurethral resection (TUR) is often followed by intravesical instillations with chemotherapy or immunotherapy.

Until now, all knowledge of disease characteristics of superficial bladder cancer (such as stage distribution, the risk of recurrences and disease progression and survival) has been based on fairly small case series which were often selective in one or more respects. In this paper, quantitative data of clinical epidemiological features of superficial bladder cancer are presented from a large case series in the Netherlands.

### Patients and methods

In the southeastern part of the Netherlands, there has been a close cooperation between urologists, pathologists and radiotherapists from 23 district hospitals, one university hospital and six radiotherapy centres since 1981 (the Dutch South-East Cooperative Urological Group). In 1983, this resulted in a consensus report on the diagnosis and treatment of patients with bladder cancer. Furthermore, it was agreed that the participating urologists would document every newly diagnosed bladder cancer patient. This documentation project started in 1983. Intake registration continued until January 1990. Follow-up registration continued until July 1991.

The following items of each patient with a primary bladder tumour were registered: date of birth, sex, date of histological confirmation, main complaint, tumour morphology, grade of differentiation (according to the WHO grading system: Mostofi, 1973), localisation, TNM classification (UICC, 1978), tumour multiplicity and intravenous urogram result. Furthermore, all participating urologists were asked to take (and document) at least four random quadrant biopsies in macroscopically normal-looking urothelium (left and right lateral wall, trigone and dome) at the time of resection of the tumour(s). The therapy to be applied was transurethral resection of the tumour (TUR) in all patients. Urologists were advised to consider adjuvant intravesical instillations with chemotherapy or BCG vaccine in the case of multiple tumours. In pT1 grade 3 patients more aggressive therapy, such as radical surgery or external or interstitial radiotherapy, would have to be considered. To detect recurrences, cystoscopy and urine cytology were used every 3 months in the first year after treatment. From the second year onwards, this check-up was performed every 6 months. Follow-up data concerning disease and life status were collected for each patient once every year.

Between 1983 and 1990, 2,805 cases were documented. In 1991, all the data in the documentation project were reviewed using the medical files. After this check, the records of 100 cases were excluded. Of these, 30 had an inverted papilloma (which was considered to be benign), 58 had recurrent instead of primary disease at first registration, five did not have TCC in the bladder but in the upper urinary tract. In the records of seven cases, there were major inconsistences, which could not be corrected with information from the medical files. Of the remaining 2,705 cases, 1,745 (64.5%) had superficial TCC. 'Superficial' is defined as tumour extension limited to the mucosa (pTa) or the lamina propria (pT1) of the bladder wall with or without carcinoma *in situ* in random biopsies. In urology practice, primary carcinoma *in situ* (pTis) is considered to be very different from pTa and pT1 tumours because of its relatively aggressive clinical behaviour. For that reason, patients with primary pTis ($n = 52$ in our series) were not evaluated in this study. Survival free of recurrence, survival free of progression and survival itself were measured from the data of histological diagnosis to the date of first recurrence, first evidence of disease progression and the date of death, respectively. Survival curves were based on the life table method, statistical significance being determined by the log rank test. The independence of host and tumour characteristics in determining survival free of recurrence and progression was evaluated multivariately using the Cox proportional hazards model (Cox, 1972).

Correspondence: L.A.L.M. Kiemeney, Department of Medical Informatics and Epidemiology, University of Nijmegen, PO Box 9101, NL-6500 HB Nijmegen, The Netherlands.
Received 30 March 1992; and in revised form 19 October 1992.

Although the case series in this documentation project is large, registration was not population based, which implies that incidence rates cannot be calculated from the project. However, nine Comprehensive Cancer Centres in the Netherlands keep population based regional cancer registries (IKL), covering a population of approximately 850,000 in the southern part of the Netherlands, has complete data on the incidence of superficial bladder cancer since 1986 (Schouten *et al.*, 1992). Information from this registry from the period 1986–1989 was used to calculate age and sex-specific incidence rates. The population based cancer registry was also used to check whether patient intake in the documentation project was selective in any way. There appeared to be hardly any difference in age, sex, stage and grade distribution between the cancer registry and our case series, indicating no under or over representation in our series.

### Results

### *Incidence*

In the southern part of the Netherlands, the total bladder cancer incidence rate per $10^5$ person-years (age-standardised to the European standard population) is 36.3 for males and 6.7 for females. Superficial bladder cancer incidence rates for males and females are 23.7 and 3.9, respectively. This accounts for 65% of the total bladder cancer incidence in males and 58% in females. The proportion of all bladder cancers diagnosed as superficial disease is higher in the younger age categories than in the older ones (Figure 1). In males, the lifetime risk of developing bladder cancer (before the age of 75 years) is 2.8%. The risk of superficial bladder cancer is 1.9%. In females, these risks are 0.5% and 0.4%, respectively.

### *Initial presentation*

In the documentation project, 1,745 patients with superficial (pTa or pT1) TCC were registered. Characteristics of this group of patients at initial presentation are listed in Table I: 32% of all the patients had a pT1 tumour; 16% of the tumours were classified with differentiation grade 3 and 29% had multiple tumours. Random biopsies of macroscopically normal-looking urothelium were taken in 1,044 patients. In 22% of these patients, dysplasia or carcinoma *in situ* was diagnosed in at least one biopsy specimen. The median age at the time of diagnosis was 67 years. There was a clear shift towards a higher disease stage with increasing age (Figure 2). In patients under the age of 40 years, pTa grade 1 or 2 disease accounted for more than 85% compared to only 52% in patients over 80 years of age. The proportion of patients diagnosed with multiple tumours was also different for these age groups: 13% *vs* 37%.

From the total group of patients, 64% were treated with transurethral resection only; additional intravesical instillations were administered in 32% of the cases and more extensive therapy was given in 4% of the cases (radiotherapy and/or cystectomy). The latter 4% were excluded from the analysis of the risks of recurrence and progression.

### *First recurrence*

The life-table (or actuarial) risk of recurrent disease after primary treatment in superficial bladder cancer was very high. Within 5 years, nearly 60% of all the cases had at least one recurrence (Figure 3). A proportion of this group of patients with a recurrence were prone to having more recurrences. The risk of recurrent disease in the first year of follow-up was 33% (95% CI: 31–35%). In the second year of follow-up, this risk was as high as 47% among the patients who had already had recurrent disease, compared to only 18% of the patients without recurrence in the first year of follow-up.

The risk of recurrent disease in superficial bladder cancer was dependent on a number of prognostic indicators. In the univariate analyses, tumour stage (pT1 *vs* pTa), degree of differentiation (grade 3 *vs* 2 *vs* 1), multiplicity (multiple *vs* solitary) and extent of the tumour (involvement of more than one bladder area *vs* 1 area) had statistically significant effects on the risk of recurrence (all log-rank tests yielded $P$ values $<0.001$). In our study, the risk of recurrence was not

**Figure 1** Age-specific incidence rates of total bladder cancer and superficial bladder cancer in males and females in the province of Limburg (NL), 1986–1989.

**Table I** Clinical characteristics at disease presentation and the therapy applied in 1,745 patients with primary superficial TCC

| | n | % |
|---|---|---|
| *Sex* | | |
| Men | 1415 | 81.1 |
| Women | 330 | 18.9 |
| *Age* | | |
| <= 39 | 46 | 2.6 |
| 40-49 | 103 | 5.9 |
| 50-59 | 295 | 16.9 |
| 60-69 | 550 | 31.5 |
| 70-79 | 546 | 31.3 |
| 80+ | 205 | 11.7 |
| *Main complaint (n = 1227)* | | |
| Haematuria | 991 | 80.8 |
| Irritative bladder symptoms | 156 | 12.7 |
| Not urological | 80 | 6.5 |
| *Stage* | | |
| pTa | 1187 | 68.0 |
| pT1 | 558 | 32.0 |
| *Grade*[a] | | |
| 1 | 669 | 38.4 |
| 2 | 793 | 45.4 |
| 3 | 283 | 16.2 |
| *Multiplicity* | | |
| Solitary | 1223 | 70.1 |
| Multiple | 510 | 29.2 |
| Unknown | 12 | 0.7 |
| *Areas involved* | | |
| Neck only | 27 | 1.5 |
| Trigone only | 55 | 3.2 |
| Posterior wall only | 123 | 7.0 |
| Right lateral wall only | 384 | 22.0 |
| Left lateral wall only | 390 | 22.3 |
| Dome only | 38 | 2.2 |
| Anterior wall only | 25 | 1.4 |
| 2 Areas | 373 | 21.4 |
| 3 Areas | 166 | 9.5 |
| >=4 Areas | 164 | 9.4 |
| *Quadrant biopsies* (*n* = 1044) | | |
| No abnormalities | 816 | 78.2 |
| Dysplasia in 1 or more areas | 142 | 13.6 |
| Carcinoma *in situ* | 86 | 8.2 |
| *Therapy* | | |
| TUR only | 1116 | 64.0 |
| TUR + instillations | 558 | 32.0 |
| (Partial) cystectomy or interstitial radiotherapy | 71 | 4.1 |

[a]In cases with different grades in one tumour, the highest grade was documented.

significantly different in male and female patients ($P = 0.56$) and in patients younger and older then 70 years of age ($P = 0.93$). The risk in patients with concomitant dysplastic abnormalities in normal-looking urothelium was only slightly higher than the risk in patients without these abnormalities: 57% and 51%, respectively ($P = 0.10$).

Even though intravesical chemotherapy or BCG was applied more frequently in patients with a poor prognostic profile, adjuvant therapy proved to be effective for preventing recurrences. In the patients who were treated with TUR alone, the 3-year risk of recurrence was 55%, whereas this risk was 49% ($P = 0.005$) in the cases treated with intravesical instillations.

We subsequently re-evaluated all the factors in a multivariate proportional hazards regression model. In this model, we also adjusted for the potential distorting effect of adjuvant intravesical chemotherapy. The results remained practically the same compared to those from the univariate analyses. Tumour stage, grade, extent and multiplicity had statistically significant independent prognostic value regarding the risk of first recurrence (see Table II). The administration of intravesical therapy reduced this risk. When analysed multivariately, however, the result of random biopsies had no significant prognostic value: the relative risk of urothelial dysplasia or CIS compared to normal urothelium was only 1.11 (95% CI: 0.89–1.39).

Patients with the most favourable prognostic profile may therefore be defined as those with a solitary pTa grade 1 tumour located in just one area. In our study cohort, 390 patients had such a favourable score on all four factors. The 3-year risk in this group of patients (20% of whom were treated with intravesical instillations) was 37%. In the prognostically least favourable group of 55 patients with multiple pT1 grade 3 tumours located in more than one area, the 3-year risk was as high as 77%, even though 69% of these patients were treated adjuvantly.

### *Progression*

In the documentation project, disease progression was defined as a shift to a higher stage category (or the development of metastases). The 3-year actuarial risk of progressive disease was 10.2% (95% CI: 8.6–11.8%). After 5 years, this risk had hardly increased: 13.3%. As is the case with the risk of recurrence, tumour stage, grade, extent and multiplicity appeared to have prognostic significance for the risk of pro-

**Figure 2** Distribution (%) of stage and grade by age category in patients with superficial TCC of the bladder.

**Figure 3** Actuarial risk (%) of recurrent disease in patients with superficial TCC of the bladder (with 95% confidence interval).

**Table II** Results of the multivariate proportional hazards regression model on the risk of first recurrence

| *Factor* | *Relative risk* | *95% Confidence interval* |
|---|---|---|
| Sex: | | |
| Male *vs* female | 1.01 | 0.84–1.21 |
| Age: | | |
| =>70 *vs* <70 | 0.92 | 0.80–1.06 |
| Tumour stage: | | |
| pT1 *vs* pTa | 1.38 | 1.16–1.65 |
| Tumour grade: | | |
| 2 or 3 *vs* 1 | 1.22 | 1.04–1.43 |
| Tumour extent: | | |
| =>2 areas *vs* 1 | 1.34 | 1.14–1.57 |
| Multiplicity: | | |
| Multiple *vs* solitary | 1.42 | 1.21–1.67 |
| Random biopsies:[a] | | |
| Dysplasia/CIS *vs* normal | 1.11 | 0.89–1.39 |
| Therapy: | | |
| Instillations *vs* TUR alone | 0.67 | 0.57–0.80 |

[a]In the model, a separate biopsy category was included for the patients from whom no biopsies were taken.

gression. Furthermore, the 3-year risk in patients older than 70 years was 12.7%, whereas this risk was only 7.4% in patients younger than 70 years of age ($P = 0.001$).

Concomitant intraurothelial dysplasia or CIS significantly increased the risk of progression. In patients with such abnormalities the risk was 21%, whereas this risk was only 7% in patients without dysplastic abnormalities ($P < 0.001$). Contrary to the effect on recurrence, intravesical instillations did not lower the risk of progression. Because the group of patients who received adjuvant therapy had a poorer prognostic profile, the risk of progression in this group was even higher than in the patients treated with TUR alone (12% *vs* 8%).

Except for tumour extent, all the factors with prognostic value in the univariate analyses retained their statistically significantly quality in the multivariate regression model.

*Survival*

The actuarial risk of dying within 5 years after diagnosis was 25% (95% CI: 23–27%) (Figure 4). This risk has to be compared to the expected risk of dying from all causes given the age and sex distribution of this group of patients. Using data from the Registration of Causes of Death from the Dutch Central Bureau of Statistics, we calculated the expected risk to be 13%. Therefore, the relative 5-year survival of patients with superficial bladder cancer was (100–25)/(100–13) = 86%. Thus, the excess risk of dying within 5 years was approximately 14%.

### Discussion

The reported distributions of disease characteristics at initial diagnosis in our patients with primary superficial TCC, may not be representative for the situation in other countries. Especially the distribution of grade and cold biopsy results may differ because there are not yet any objective criteria available to enable all pathologists to classify urothelium specimens in a reproducible manner (Jordan *et al.*, 1987; Pauwels *et al.*, 1988; Richards *et al.*, 1991). Although the stage distribution is believed to be a better measure for comparison, different interpretations of the pT category by different pathologists are also possible (Abel *et al.*, 1988*a*; Kurth *et al.*, 1989; Parmar *et al.*, 1989; Herr & Jakse, 1991). Another factor which very often influences the distribution of disease characteristics is the inclusion of patients with recurrent instead of primary disease. Over the past 15 years, it is likely that an increasing number of 'papillomas' have been classified as papillocarcinomas (until 1978, the UICC listed only the category pT1 for superficial bladder cancer). For this reason, comparison with other case series is only worthwhile if these series were documented fairly recently. In a recent study by Abel, 107 (62.6%) out of the 171 cases with bladder cancer had superficial disease at presentation (Abel *et al.*, 1988*b*). Of these, 71% were classified as pTa. From the total group, 60.7% (compared to 70.1% in our study) had solitary tumours. Grade 1, 2 and 3 accounted for 6.5%, 85.0% and 8.4% of all the tumours, respectively. This distribution, which is very different from the finding in our study, illustrates the need for better reproducible methods for the assessment of certain indicators used for prognosis. In a recent Danish study, 61% of 500 bladder cancer cases had superficial disease, of whom 69% had stage pTa (Wolf *et al.*, 1987). These numbers are very similar to those in our study.

**Figure 4** Actuarial survival (%) in patients with superficial TCC of the bladder (with 95% confidence interval) compared to the Dutch population.

In the Danish case series, 7.9% of the pTa/pT1 patients had carcinoma *in situ* in the cold-cup biopsy specimens taken at the first presentation of disease. Another 15.4% showed atypia grade 2. In a study by Flamm and Dona, CIS was found in the quadrant biopsy specimens in 6% of 216 patients. Dysplasia was found in 18% (Flamm & Dona, 1989). In our study, the corresponding percentages were 8.2 and 13.6, respectively. In a recent study by Solsona *et al.* (1991), 48 out of the 306 patients with superficial bladder tumours had associated carcinoma *in situ*, but this high number was caused by the inclusion of random area as well as suspicious area biopsies.

Nearly 60% of all the patients in our study had at least one recurrence within 5 years; most of them within 2 years (2-year recurrence risk: 45%). In fact, the recurrence risk in superficial bladder cancer is so high that (as opposed to other cancer sites) a second occurrence of TCC in the bladder is always interpreted as a recurrence, although this is theoretically incorrect (Abel, 1988). The recurrence risk is dependent on a number of prognostic factors (Abel, 1988; Lum & Torti, 1991). In our case series, we studied the effect of seven prognostic factors and found that tumour stage, grade, extent and multiplicity were statistically significant prognostic indicators. Using these indicators, it may be possible to discern groups of patients with very different risks of recurrence. This, however, does not mean that it will be possible to predict the risk of individual patients fairly accurately. After all, for an individual patient there are only two possible outcomes: either he suffers a recurrence or he does not. Until we are able to differentiate all superficial bladder cancer patients into one group with a 100% recurrence risk and one group with a 0% recurrence risk, predictions for individuals will always be inaccurate (Levine *et al.*, 1991). The finding in our project that even the best prognostic group still had a 3-year recurrence risk of 37%, rather than 0%, shows that the inaccurate measurement of prognostic indicators together with biological variablility inevitably leads to inaccurant predictions. Although we have a number of highly significant prognostic indicators for the risk of recurrence, apparently we do not have enough of these indicators yet.

Superficial bladder cancer patients have a relatively high survival rate. In our study, the 5-year survival was 75%, compared to 87% for the Dutch population adjusted for age, sex and calendar period. This finding is very similar to the 88% 5-year survival rate for early stage bladder cancer (adjusted for normal life-expectancy) in the USA (American Cancer Society, 1991). In a recent study in the United Kingdom, the 5-year survival in 150 pTa and 85 pTa patients was 80 and 69%, respectively (Gulliford *et al.*, 1991). Flamm and Havelec (1990) found a tumour-related mortality rate of 12.5% in 345 patients with primary superficial TCC treated with TUR and intravesical instillations.

Despite the fact that the excess mortality in superficial bladder cancer is small, it may be asked why there is any excess mortality at all. Theoretically, it is possible that some of the superificial bladder cancers were already higher stage cancers at initial diagnosis. It is not exceptional for the pathologist to receive a resection specimen that does not contain any muscle tissue. Thus, some T2 tumours (with a poorer prognosis) may have been staged as pT1 tumours. In the surveillance programme of the USA National Bladder Cancer Collaborative Group, for example, there was no muscle tissue present in the specimens of 40 out of 95 tumours classified as pT1 (Cutler *et al.*, 1982). By contrast, in a recent study of Abel *et al.* (1988*a*), muscle tissue was present in 95% of the pTa/pT1 biopsy specimens. Unfortunately, comparable information was not available in our project.

Another possible explanation for excess mortality is undertreatment. Especially multiple high grade tumours which extend into the lamina propria are often seen to progress to higher stage disease (Cutler *et al.*, 1982; Pocock *et al.*, 1982; Heney *et al.*, 1983). But until now, this knowledge has not led to a consensus policy of treating all 'high risk' tumours with (at least) intravesical instillations. According to our data, a surprisingly high number (26%) of patients with multiple pT1 grade 3 tumours were treated with TUR only. Nevertheless, it is dubious whether intravesical instillations prevent progression. Intravesical chemotherapy will usually decrease the rate and number of recurrences, but according to many authors it does not necessarily alter the ultimate outcome of the disease (Heney, 1988; Flamm & Havelec, 1990; Newling, 1990; Soloway *et al.*, 1990; Vögeli & Ackermann, 1990). Our data support the observation of these authors. As Table III illustrates, intravesical instillations

**Table III** Influence of the use of intravesical instillations for the treatment of the primary tumour on the 5-year actuarial risk of progressive disease[1]

| | *5-year risk of progressive disease (%)* | | |
|---|---|---|---|
| *n = 1674* | *TUR only* | *Intravesical instillations* | *Log-rank P value* |
| Sex | | | |
| Male | 11.1 (*n* = 902) | 14.7 (*n* = 449) | 0.05 |
| Female | 11.0 (*n* = 214) | 19.6 (*n* = 109) | 0.06 |
| Age | | | |
| <70 | 9.1 (*n* = 634) | 14.5 (*n* = 314) | 0.01 |
| =>70 | 13.6 (*n* = 482) | 17.0 (*n* = 244) | 0.29 |
| Stage | | | |
| pTa | 8.3 (*n* = 840) | 10.1 (*n* = 339) | 0.66 |
| pT1 | 19.3 (*n* = 276) | 24.7 (*n* = 219) | 0.07 |
| Grade | | | |
| 1 | 4.1 (*n* = 531) | 7.7 (*n* = 137) | 0.29 |
| 2 | 15.9 (*n* = 495) | 12.0 (*n* = 277) | 0.24 |
| 3 | 25.3 (*n* = 90) | 32.2 (*n* = 144) | 0.29 |
| Multiplicity | | | |
| Solitary | 8.8 (*n* = 856) | 10.4 (*n* = 325) | 0.27 |
| Multiple | 18.6 (*n* = 254) | 24.1 (*n* = 229) | 0.32 |
| Random biopsies | | | |
| Normal | 10.1 (*n* = 533) | 7.0 (*n* = 260) | 0.40 |
| Dysplasia/CIS | 20.2 (*n* = 74) | 26.7 (*n* = 134) | 0.30 |

[a]Progressive disease is defined as any shift to a higher stage category or the development of metastases.

seem to increase rather than decrease the 5-year risk of progressive disease. However, this finding was caused by the fact that more patients with a relatively poor prognosis received adjuvant therapy. In a proportional hazards model with sex, age, tumour stage, grade, multiplicity, random biopsy result and therapy, the relative risk of TUR-only *vs* intravesical instillations was 1.0 (95% CI: 0.7–1.3). Nevertheless, even though analysed multivariately, this finding has to be interpreted with caution because our study was not initiated to study therapy effects and therefore was not randomised. Furthermore, most of the patients in our study who were treated adjuvantly, received intravesical instillations with Doxorubicin or Mitomycin-C. Intravesical immunotherapy with Bacillus Calmette-Guérin RIVM, a strain produced by the Dutch National Institute of Public Health and Environmental Hygiene, was applied less frequently. Two recent studies give some indication that BCG may be more effective in preventing progression than the chemotherapeutical agents (Herr *et al.*, 1988; Eure *et al.*, 1992). Also, at the present time t = n EORTC phase II study is going on in which a sequential combination of intravesical chemotherapy with Mitomycin-C and intravesical immunotherapy with BCG is evaluated in recurrent superficial TCC of the bladder. Possibly, in the near future such a combination regimen may appear to be effective in delaying or preventing disease progression.

In summary, we can conclude that even superficial TCC, as a distinct subgroup of bladder cancer, is a heterogeneous disease with an unpredictable clinical course. Even though some important indicators for future recurrences and progressive disease can be identified (such as tumour stage, grade and multiplicity), it remains a challenge to find more and stronger prognosticators which can more accurately predict the disease outcome in individual patients. Only then will it be possible to treat patients with a poor prognosis more aggressively and to avoid overtreatment of patients with a fairly good prognosis.

We thank Mrs Rie Speyers-van Doremalen and Ms Marjorie de Kok for data management.

This work was supported by grants from the Comprehensive Cancer Centres IKO, IKZ and IKAST.

### References

ABEL, P.D., HENDERSON, D., BENNETT, M.K., HALL, R.R. & WILLIAMS, G. (1988*a*). Differing interpretations by pathologists of the pT category and grade of transitional cell cancer of the bladder. *Br. J. Urol.*, **62**, 339–342.

ABEL, P.D., HALL, R.R. & WILLIAMS, G. (1988*b*). Should pT1 transitional cell cancers of the bladder still be classified as superficial? *Br. J. Urol.*, **62**, 235–239.

ABEL, P.D. (1988). Prognostic indices in transitional cell carcinoma of the bladder. *Br. J. Urol.*, **62**, 103–109.

AMERICAN CANCER SOCIETY (1991). *Cancer Facts and Figures 1991*. ACS: Atlanta.

COX, D.R. (1972). Regression models and life-tables. *J. R. Stat. Soc.*, **34**, 187–220.

CUTLER, S.J., HENEY, N.M. & FRIEDELL, G.H. (1982). Longitudinal study of patients with bladder cancer: factors associated with disease recurrence and progression. In *Bladder Cancer*, Bonney, W.W. & Prout, G.R. (eds). pp. 35–46. Williams & Wilkins: Baltimore.

EURE, G.R., CUNDIFF, M.R. & SCHELLHAMMER, P.F. (1992). Bacillus Calmette Guérin therapy for high risk stage T1 superficial bladder cancer. *J. Urol.*, **147**, 376–379.

FLAMM, J. & DONA, ST. (1989). The significance of bladder quadrant biopsies in patients with primary superficial bladder carcinomas. *Eur. Urol.*, **16**, 81–85.

FLAMM, J. & HAVELEC, L. (1990). Factors affecting survival in primary superficial bladder cancer. *Eur. Urol.*, **17**, 113–118.

GULLIFORD, M.C., PETRUCKEVITCH, A. & BURNEY, P.G.J. (1991). Survival with bladder cancer, evaluation of delay in treatment, type of surgeon, and modality of treatment. *Br. Med. J.*, **303**, 437–440.

HENEY, N.M., AHMED, S., FLANAGAN, M.J., FRABLE, W., CORDER, M.P., HAFERMANN, M.D. & HAWKINS, I.R. (1983). Superficial bladder cancer: progression and recurrence. *J. Urol.*, **130**, 1083–1086.

HENEY, N.M. (1988). Intravesical chemotherapy: how effective is it? *Urol.*, **31** (Suppl), 17–19.

HERR, H.W. (1991). Transurethral resection and intravesical therapy of superficial bladder tumors. *Urol. Clin. North. Am.*, **18**, 525–528.

HERR, H. & JAKSE, G. (1991). pT1 bladder cancer. *Eur. Urol.*, **20**, 1–8.

HERR, H.W., LAUDONE, V.P., BADALAMENT, R.A., OETTGEN, H.G., SOGANI, P.C., FREEDMAN, B.D., MELAMED, M.R. & WHITMORE, W.F. Jr (1988). Bacillus Calmette-Guérin therapy alters the progression of superficial bladder cancer. *J. Clin. Oncol.*, **6**, 1450–1455.

JORDAN, A.M., WEINGARTEN, J. & MURPHY, W.M. (1987). Transitional cell neoplasms of the urinary bladder. Can biologic potential be predicted from histologic grading? *Cancer*, **60**, 2766–2774.

KURTH, K.H., SCHROEDER, F.H., DEBRUYNE, F.M.J., SENGE, T., PAVONE-MACALUSO, M., DE PAUW, M., TEN CATE, F. & SYLVESTER, R. (1989). Long-term follow-up in superficial transitional cell carcinoma of the bladder: prognostic factors for time to first recurrence, recurrence rate, and survival. *Prog. Clin. Biol. Res.*, **303**, 482–490.

LEVINE, M.N., BROWMAN, G.P., GENT, M., ROBERTS, R. & GOODYEAR, M. (1991). When is a prognostic factor useful? A guide for the perplexed. *J. Clin. Oncol.*, **9**, 348–356.

LUM, B.L. & TORTI, F.M. (1991). Adjuvant intravesicular pharmacotherapy for superficial bladder cancer. *J. Natl Cancer Inst.*, **83**, 682–694.

MOSTOFI, F.K. (1973). *Histological Typing of Urinary Bladder Tumours*. International histological classification of tumours (ed 10). WHO: Geneva.

NEWLING, D. (1990). Intravesical therapy in the management of superficial transitional cell carcinoma of the bladder: the experience of the EORTC GU group. *Br. J. Cancer*, **61**, 497–499.

PARMAR, M.K.B., FREEDMAN, L.S., HARGREAVE, T.B. & TOLLEY, D.A. (1989). Prognostic factors for recurrence and followup policies in the treatment of superficial bladder cancer: report from the British Medical Research Council subgroup on superficial bladder cancer (urological cancer working party). *J. Urol.*, **142**, 284–288.

PAUWELS, R.P.E., SCHAPERS, R.F.M., SMEETS, A.W.G.B., DEBRUYNE, F.M.J. & GERAEDTS, J.P.M. (1988). Grading in superficial bladder cancer. 1. Morphological criteria. *Br. J. Urol.*, **61**, 129–134.

POCOCK, R.D., PONDER, B.A.J., O'SULLIVAN, J.P., IBRAHIM, S.K., EASTON, D.F. & SHEARER, R.J. (1982). Prognostic factors in non-infiltrating carcinoma of the bladder: a preliminary report. *Br. J. Urol.*, **54** 711–715.

RICHARDS, B., PARMAR, M.K.B., ANDERSON, C.K., ANSELL, I.D., GRIGOR, K., HALL, R.R., MORLEY, A.R., MOSTOFI, F.K., RISDON, R.A. & USCINSKA, B.M. (1991). Interpretation of biopsies of 'normal' urothelium in patients with superficial bladder cancer. *Br. J. Urol.*, **67**, 369–375.

SCHOUTEN, L.J., VAN DEN BRANDT, P.A. & JAGER, J.J. (1992). Cancer incidence in the province of Limburg, the Netherlands. *Eur. J. Cancer*, **28A**, 1752–1755.

SOLOWAY, M.S., MURPHY, W.M., JOHNSON, D.E., FARROW, G.M., PAULSON, D.F. & GARNICK, M.B. (1990). Initial evaluation and response criteria for patients with superficial bladder cancer. Report of a workshop. *Br. J. Urol.*, **66**, 380–385.

SOLSONA, E., IBORRA, I., RICÓS, J.V., MONROS, J.L., DUMONT, R., CASANOVA, J. & CALABUIG, C. (1991). Carcinoma in situ associated with superficial bladder tumor. *Eur. Urol.*, **19**, 93–96.

UICC (1978). *TNM Classification of Malignant Tumours* (ed 3). International Union against Cancer: Geneva.

VÖGELI, T. & ACKERMANN, R. (1990). When does superficial bladder cancer resist intravesical therapy? *Sem. Urol.*, **8**, 248–253.

WOLF, H., ROSENKILDE OLSEN, P., FISCHER, A. & HOJGAARD, K. (1987). Urothelial atypia concomitant with primary bladder tumor. *Scand. J. Urol. Nephrol.*, **21**, 33–38.